# How Many Is Enough? - Challenges of Multinucleated Cell Division in Malaria Parasites

**DOI:** 10.3389/fcimb.2021.658616

**Published:** 2021-05-07

**Authors:** Caroline S. Simon, Vanessa S. Stürmer, Julien Guizetti

**Affiliations:** Centre for Infectious Diseases, Heidelberg University Hospital, Heidelberg, Germany

**Keywords:** malaria, *Plasmodium*, fungi, cell division, multinucleated, mitosis, polyploidy

## Abstract

Regulating the number of progeny generated by replicative cell cycles is critical for any organism to best adapt to its environment. Classically, the decision whether to divide further is made after cell division is completed by cytokinesis and can be triggered by intrinsic or extrinsic factors. Contrarily, cell cycles of some species, such as the malaria-causing parasites, go through multinucleated cell stages. Hence, their number of progeny is determined prior to the completion of cell division. This should fundamentally affect how the process is regulated and raises questions about advantages and challenges of multinucleation in eukaryotes. Throughout their life cycle *Plasmodium* spp. parasites undergo four phases of extensive proliferation, which differ over three orders of magnitude in the amount of daughter cells that are produced by a single progenitor. Even during the asexual blood stage proliferation parasites can produce very variable numbers of progeny within one replicative cycle. Here, we review the few factors that have been shown to affect those numbers. We further provide a comparative quantification of merozoite numbers in several *P. knowlesi* and *P. falciparum* parasite strains, and we discuss the general processes that may regulate progeny number in the context of host-parasite interactions. Finally, we provide a perspective of the critical knowledge gaps hindering our understanding of the molecular mechanisms underlying this exciting and atypical mode of parasite multiplication.

## Introduction

Cell types containing multiple genome copies emerged independently on various branches of the eukaryotic tree of life (Comai, 2005), which suggests convergent evolution of this trait in order to accommodate very different adaptive needs (Campbell et al., 2016). Multinucleated cells are prominent in filamentous fungi (Roper et al., 2013; Mela et al., 2020). In some cases those induce polyploidy as an adaptation to stress (Selmecki et al., 2010; Selmecki et al., 2015) or individual nuclei can be degraded as a nutrient source under starvation conditions (Shoji et al., 2010). Having multiple genome copies within one cell can further provide a buffering function, allowing diversification of the genome by structural variation or single nucleotide polymorphisms, without suffering deleterious consequence of those mutations. Hence, filamentous fungi establish long-lived compartments in which genetically divergent nuclei can co-exist, compete and even undergo horizontal gene transfer by fusions of nuclei (Mela et al., 2020). The range of unicellular organisms being multinucleated stretches from yeasts to algae (Coneva and Chitwood, 2015; Mitchison-Field et al., 2019). A striking example is *Caulerpa* spp., a single-celled alga that can reach a length of meters and contains myriads of nuclei (Jacobs, 1994). Within multicellular organisms the mammalian placenta, bones, and muscles are known to form multinucleated cell types (Mintz and Baker, 1967; Marks, 1983; Mi et al., 2000; Płachno and Światek, 2011). In plants, appearance of multinucleated cells has been associated with the increase of cell size and thereby growth of the organism as a whole (Płachno and Światek, 2011; Coneva and Chitwood, 2015). In those cases multinucleation is necessary to ensure the scaling of genome copies with cytoplasmic volume, which facilitates homeostatic protein expression or gene dosage (Marguerat and Bähler, 2012) (see also Machado et al. in this issue). Plants can sometimes even form giant cells containing up to 100 nuclei, when infected by nematodes (Favery et al., 2016). Interestingly, an organism with a more *Plasmodium*-related multiplication mode is the free-living unicellular marine *Ichthyosporea*, a putative predecessor of multicellular animals (Ondracka et al., 2018). It generates up to 128 nuclei by synchronous divisions before completing daughter cell formation and releasing them through bursting. We, however, argue that malaria parasites use multinucleation in very different adaptive context. When transmitting between the mosquito vector and the human host, malaria parasites encounter important population bottlenecks and react to those steep decreases of population size by phases of extensive proliferation. *Plasmodium* parasites undergo multiple cycles of nuclear division, while cytokinesis only occurs prior to egress from the host cell. Hence, the final number of nuclei determines how many daughter cells will emerge from a single progenitor. The plasticity of *Plasmodium* spp. division with respect to the total number of daughter cells produced within one replicative cycle is remarkable (Matthews et al., 2018). In this review we i) summarize the importance of multinucleation, ii) discuss how progeny number might be regulated, and iii) define critical cellular parameters that remain to be assessed to better understand this process.

## Multinucleation, Polyploidy, Syncytium, and Coenocyte

Since the terminology around multi-genome containing cells is sometimes contradictory, we want to define our usage of those terms. Ploidy refers to the number of chromosomes sets per cell. In a normally haploid cell, any genome copy number higher than two, which is the post-replicative state, would be considered polyploid. Multinucleation refers to the number of physically separated nuclei within a common cytoplasm. Polyploidy and multinucleation are not per se mutually exclusive. In principle a multinucleated cell can arise by two ways. If multinucleation is a result of cell-cell fusion, which has not been reported in malaria parasites, it is referred to as syncytium (Daubenmire, 1936). Multiple rounds of nuclear division that occur within a common cytoplasm cause the formation of a coenocyte.

## Progeny Numbers in *Plasmodium* Species Display Remarkable Plasticity

**Figure 1:**
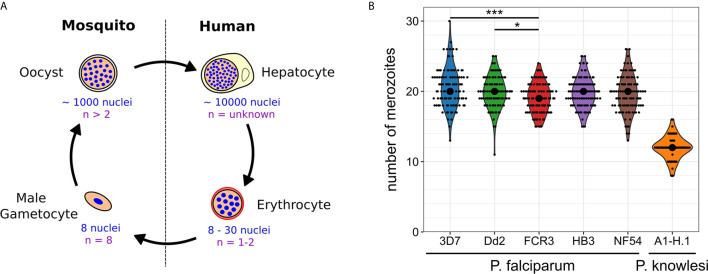
Nuclear and genome copy numbers throughout *Plasmodium* spp. life cycle stages. **(A)** Schematic depiction of approximate total number of nuclei produced during the proliferative life cycle stages of *P. falciparum* as well as the highest observed ploidy (n) or genome copy number within individual nuclei. **(B)** Number of merozoites produced in *P. falciparum* and *P. knowlesi*. To count merozoites, late stage parasites were enriched by magnetic purification or Nycodenz density gradient. Parasites were incubated with the cyclic GMP-dependent protein kinase (PKG) inhibitor ML10 (25 nM in *P. falciparum* strains; 125 nM in *P. knowlesi*) for 3 h to prevent egress, increasing the percentage of post-mitotic parasites. After preparation of blood smears parasites were fixed with 4% paraformaldehyde for 20 min at 37°C. Before imaging, cells were stained with Hoechst. Microscopy was performed on a Leica TCS SP8 scanning confocal microscope with Lightning (LNG) module. LNG enables automated adaptive deconvolution after acquisition resulting in super-resolution images. For each *Plasmodium* strain, 100 cells were imaged. Merozoites were counted in a single-blind mode by three independent researchers using Fiji. The mean merozoite number for each individual cell was taken to create the violin plot. The centered black dot represents the median. High density of nuclei in *P. falciparum* made it difficult to assess the definitive final number of nuclei. This counting error might contribute to the presence of many uneven numbers in *P. falciparum* strains, while in *P. knowlesi* almost exclusively even numbers were counted. For statistical analysis, a Welch-ANOVA test was performed, and individual lines were compared with a *post hoc* Games-Howell test using R studio. For *P. falciparum* strains, FCR3 and 3D7 (p = 2.4x10^-4) as well as FCR3 and Dd2 (p = 4.2x10^-2) showed significant differences.

*Plasmodium* spp. experience polyploid as well as multinucleated stages throughout their life cycle (Francia and Striepen, 2014; Gubbels et al., 2020). The timing of those developmental stages differs significantly and the number of progeny generated by a single progenitor cell varies over three orders of magnitude (**Figure 1A**). When a mosquito takes up a *Plasmodium*-infected blood meal it triggers conversion of male gametocytes into gametes. Activated gametocytes undergo three rounds of DNA replication yielding a polyploid nucleus with 8 chromosome sets, which are then packaged into male gamete flagella (Sinden et al., 1978). This process is completed in less than 15 min in *P. berghei* making it an extreme example of fast DNA replication (Fang et al., 2017; Matthews et al., 2018). The fact that about 60% of gametes are malformed or lack nuclei highlights a potential trade-off between speed and accuracy (Sinden et al., 2010). Once male and female gametes have fused and undergone meiotic division, they embed themselves in the mosquito midgut wall, where oocysts develop. An oocyst takes a few days to mature and can produce up to thousand sporozoites (Beier, 1998; Vaughan, 2007). Polyploidy in this stage is indicated by the presence of multipolar spindles (Schrével et al., 1977; Spreng et al., 2019). When an infected mosquito takes a blood meal it injects sporozoites into the host skin. Those find their way to liver cells inside which tens of thousands of merozoites are produced from a single infected hepatocyte within less than a week (Shortt et al., 1951; Prudêncio et al., 2006; Sturm et al., 2006). Merozoites are then released into the blood where they start the replicative cycles that cause pathogenesis. Once they invade a red blood cell, parasites undergo asynchronous divisions, followed by a final synchronous one. The nuclei alternate between haploid and diploid stages, while polyploidy is usually not observed (Janse et al., 1986; Klaus et al., 2021). The total number of daughter cells resulting from these nuclear division cycles does not only vary within a population but also between different *P. falciparum* strains. In the Dd2 strain between 8 and 28 merozoites have been counted with an average of 18, while HB3 had an average of 15 (Reilly et al., 2007). In 3D7 average numbers of 22 have been reported (Dorin-Semblat et al., 2008; Garg et al., 2015). In newly isolated Ghanaian parasite strains, values between 16-24 were found, although those were only measured after six months of continuous *in vitro* culture and once multiplication rates had significantly increased (Stewart et al., 2020). The counted number of merozoites can, however, vary significantly depending on the used visualization method (Garg et al., 2015). Hence, we developed an optimized merozoite counting protocol involving HyVolution-based super-resolution microscopy and applied it to commonly studied *P. falciparum* strains and laboratory-adapted *P. knowlesi* (**Figure 1B**). Contrary to our expectations, this yielded a median of 19 or 20 merozoites for all analyzed *P. falciparum* strains. Statistical difference was only found between the 3D7, Dd2, and FCR3 strains although their mean values barely deviated by more than 1 merozoite. In *P. knowlesi* we counted significantly less merozoites, which associates with the shorter cell cycle duration of about 24 hours (while *P. falciparum* takes about 48 hours). The rodent malaria parasites, which also follow a 24 hour cycle, produce about 12 merozoites in the case of *P. berghei* (Mancio-Silva et al., 2017), but only around 6 in *P. chabaudi* (Birget et al., 2019). The convergence of average merozoite numbers for *P. falciparum* strains potentially hints at the importance of growth conditions rather than of genetic or epigenetic diversity. Nevertheless, the distribution of numbers remains wide reaching from 11 to 30. What causes this variability in progeny number and if and how the parasite counts its nuclei is unknown.

## Putative Roles of Multinucleated and Polyploid Cell Stages in *Plasmodium* Species

Contrary to fungi or plants, the size of the malaria-causing parasite is restricted by its host cell and the duration of a specific multinucleated or polyploid stage is limited by the cyclical nature of the parasite’s lifestyle. Nevertheless, genome diversification *via* mitotic recombination is frequent in *Plasmodium* blood stages (Claessens et al., 2014). This effect is, however, concentrated within the clonally variant gene families mostly located at the telomeres, while the core genome is surprisingly stable (Bopp et al., 2013). Recombination events could be augmented in the polyploid mosquito stages where more sister chromatid arms can come in contact with each other. It is, nonetheless, conceivable that generating many nuclei could provide a buffering function for mutations as described in fungi, facilitating a diversified progeny that can yield better adapted offspring. Unlike in most organisms listed above, the number of generated nuclei in *Plasmodium* directly predicts the number of progeny emerging from an infected cell. Hence, a more manifest purpose of multinucleation is reaching optimal multiplication rates to generate a sufficiently high parasite concentration in the blood, which ultimately increases the probability of transmission upon mosquito bite. Blood-stage cycles of *Plasmodium* spp. always are multiples of 24 hours even though parasites cultured *in vitro* can slightly deviate from that (Reilly et al., 2007). This suggests a strong incentive for the parasite to synchronize its growth with the host circadian cycle (Rijo-Ferreira et al., 2020; Smith et al., 2020). Whether there might also be a “safety in numbers”-effect due to the synchronous burst of infected cells and release of huge numbers of merozoites remains to be investigated. We speculate that the parasite uses multinucleation to convert the maximal amount of red blood cell material into merozoites within the given cycle time. This tendency to favor speed over accuracy is underlined by the apparent loss of canonical cell cycle checkpoints involved in error correction such as the spindle assembly and DNA damage checkpoints. Coherently, this lack of cell cycle checkpoints has also been documented in particularly fast growing fungal species, such as *Hanseniaspora* (Steenwyk et al., 2019) and during the first rapid divisions of zebrafish embryos (Zhang et al., 2015). What regulates the final number of nuclei in malaria parasites is unknown, but general principles, supported by a few studies done in blood stages, can be discussed.

## Final Number of Merozoites Could be Predefined by a Counting Mechanism

**Figure 2:**
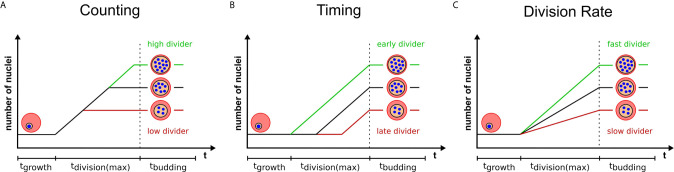
Three models highlighting variables that can contribute to progeny number. Variation in progeny number can be explained by changes in several cellular parameters. Relative number of nuclei are plotted against the total duration of blood stage development, which is divided in growth, division, and budding phase. The longest division time is marked, which starts with the first and ends with the last nuclear division. Time axes are neither to scale nor proportional. Three models provide a visual representation of changing a single determinant which limits the final progeny number and explain the variability within a single parasite population. Extrinsic or intrinsic factors can contribute to either an increase (green) or a decrease (red) of progeny number by **(A)** influencing the pre-set target number (counting), **(B)** initiating division at different times (timing) or **(C)** altering the rate of division of individual parasites.

Multinucleated parasites have to regulate the transition from nuclear division to daughter cell formation. Mechanisms that measure system size before concluding cell division have already been proposed for model organisms (Facchetti et al., 2017; Brownlee and Heald, 2019; Jones et al., 2019). Analogously, nuclear divisions could be limited by a counting mechanism that initiates cytokinesis, or the last synchronous round of division, once a target number of nuclei is reached (**Figure 2A**). The variability of progeny number within a single parasite strain argues against precise counting. A broader distribution of merozoite numbers could, however, present a competitive advantage. In a bet-hedging strategy the parasite would thereby generate sub-populations, which are better adapted to changing conditions e.g. low merozoite numbers during dropping nutrient levels in the blood (Mancio-Silva et al., 2017). If this is the case merozoite number might be an inherited trait, which predicts that parasites with a high nuclear number produce offspring that also generate high numbers. How the parasite would know how many nuclei there are and how such a mechanism would function is completely speculative. It could comprise an essential division factor available only in limiting amounts and potentially linked to extrinsic resources as discussed below. A factor that more overtly limits nuclear number is the centrosome (Arnot et al., 2011). Each round of division precludes centrosome duplication to form the poles between which chromosomes will be segregated (Simon et al., 2021). Transient localization of the aurora kinase PfArk1 to the centrosome just prior to its duplication has been described (Reininger et al., 2011). Treatment with a canonical aurora kinase inhibitor, hesperadin, causes a reduction in merozoite number, but also the appearance of resistance mutation in the PfNek1 kinase suggesting an epistatic interaction with PfArk1 (Morahan et al., 2020). The centrosome further represents the site where the transition between the nuclear division and daughter cell formation is organized in *T. gondii* (Chen and Gubbels, 2015; Suvorova et al., 2015). This is achieved by a layered centrosome structure into an inner and outer core, which can independently regulate division and budding cycles. Hence, the depletion of the transcription factor TgAP2IX-5, which acts downstream of centrosome duplication, but is required for the control of daughter cell formation can lead to *T. gondii* parasites harboring multiple nuclei (Khelifa et al., 2021).

## Final Number of Merozoites Could be Regulated by Cell Cycle Timing

Alternatively to counting, nuclear number could be defined by a timing mechanism (Facchetti et al., 2017; Jones et al., 2019) i.e. the duration of the division phase (**Figure 2B**). Indeed, several conditions have been described that delay schizogony onset (Babbitt et al., 2012; van Biljon et al., 2018; Prior et al., 2020). Time lapse imaging shows that the length of schizogony can vary from 12 to 16 hours (Klaus et al., 2021). If time limits the number of progeny, then a linear correlation between these two factors should be observable. Although *Plasmodium* spp. have no conserved “clock” genes like mammalians, they seem to possess pathways that regulate cell cycle length (Reilly Ayala et al., 2010; Rijo-Ferreira et al., 2020; Smith et al., 2020). Low nutrient levels or anemic host conditions also increase duration of the entire intraerythrocytic development cycle (Babbitt et al., 2012; Birget et al., 2019) but if these factors are influencing schizogony length is still unclear. Two kinases that have been reported to be involved in regulation of cell cycle length are PfPK7 and Pfcrk-5 (Dorin-Semblat et al., 2008; Dorin-Semblat et al., 2013). Knock-out lines for both proteins, however, exhibited longer asexual cycles and fewer nuclei per schizont suggesting merozoite release to be independent of a fixed progeny number and supporting the notion of a timer. The PfPK7 phosphoproteome further suggests implication of multiple cell-cycle associated kinases that could influence timing (Pease et al., 2018). Several other kinases associated with blood stage replication, such as Crk4, a master regulator for S-phase initiation (Ganter et al., 2017), have been described (Carvalho et al., 2016; Adderley et al., 2021). For male gametogenesis, which is activated from a G_0_-like phase upon mosquito uptake, actual signaling cascades are investigated (Invergo et al., 2017). Calcium is a secondary messenger activating CDPK4, which acts upstream of many division processes by e.g. phosphorylating the CDK-related kinase Crk5 (Fang et al., 2017). Interfering with Crk5 function by either knock down or depletion of its associated cyclin-like protein SOC2 reduces ploidy and prevents reorganization of mitotic microtubules (Balestra et al., 2020). How additional kinase pathways influence progeny number throughout the life cycle is still under investigation (White and Suvorova, 2018).

## Effect of Nuclear Division Rates

The two proposed models are very simplified in that they assume a constant nuclear division rate. But different progeny number could just as well be the result of variable division rates, while the duration of schizogony would remain equal (**Figure 2C**). Any combination of division rate and schizogony duration is also conceivable. If, however, counting is the dominant source of variability, parasites with increased division rates would just reach the predefined final number of nuclei earlier and no clear correlation between division rates and merozoite numbers should be observed.

## Influence of Extrinsic Factors on Progeny Number

Regulation of progeny number could also be influenced by extrinsic host factors. In this case the parasite would adapt its multiplication depending on the presence of certain nutrients or stress factors. Inducing anemia in mice by phenylhydrazine treatment, prior to *P. chabaudi* infection led to increase of merozoite number from 6-7 to 7-8, depending on the strain (Birget et al., 2019). Sirtuins, like Sir2, are considered metabolic sensors depending on NAD+ levels to activate their histone deacetylation activity. Sir2a in *P. falciparum* is not only involved in antigenic variation (Merrick et al., 2010; Petter et al., 2011), but its knock-out also causes increase of ribosomal RNA levels. Surprisingly, this correlates with accelerated growth, which is likely caused by the increased number of merozoites per schizont (Mancio-Silva et al., 2013). A more direct link between nutrition and progeny number was, however, demonstrated in a study infecting calorie-restricted mice with *P. berghei* parasites (Mancio-Silva et al., 2017). This causes them to produce less merozoites, a phenotype that can be rescued by supplementation with Glucose. Importantly, *Plasmodium* requires KIN kinase to regulate merozoite number in response to host starvation demonstrating the presence of a non-canonical nutrient sensing pathway. The complete depletion of Glucose from the medium, however, induces rapid death of *P. falciparum* parasites (Babbitt et al., 2012). A more subtle effect is caused by starvation from the essential amino acid isoleucine. If removed prior to schizogony a dormancy-like state is induced, which can be rescued up to 72h later (McLean and Jacobs-Lorena, 2020). Coherently, isoleucine has been postulated as one of the key factors sensed by the parasite for synchronization (Prior et al., 2020). A similar reversible arrest can be induced by addition of DL-α-difluoromethylornithine, which prevents synthesis of polyamines (van Biljon et al., 2018). Another potentially limiting resource that has been discussed, especially in the context of liver stage development, are lipids and the fatty acid synthesis pathway (Tarun et al., 2009). Although the effect on progeny number has not been quantified in most of those latter studies, they, nevertheless, highlight that the parasite can adapt its cell cycle to host cues by inducing something that could be characterized as schizogony-entry checkpoint.

## Correlation of Cell Size and Nuclear Number

An indirect way through which an extrinsic factor could affect nuclear number is through regulation of cell growth. Eukaryotic cells have a constant nucleo-to-cytoplasmic volume ratio (N/C-ratio) and an exception to this rule has not been found to date (Cantwell and Nurse, 2019). Our unpublished data suggest that this remains valid for *P. falciparum*. A simplistic model would posit that as the cytoplasmic volume increases the parasite forms more nuclei keeping the N/C-ratio constant. It is noteworthy, that blood stage parasite volume only increases by a factor of about seven (Waldecker et al., 2017), while nuclear numbers get clearly higher (**Figure 1**). Nevertheless, nuclear volume can be altered by chromatin compaction. Indeed, when comparing maximal intensities of a DNA stain like Hoechst, it is clearly higher in rings versus trophozoites, which have a greater cell and nuclear volume. Similarly, nuclear surface in late schizonts seems to decrease, while number of nuclei increases. More detailed analysis of the correlation between those parameters would clearly benefit from the discovery of a nuclear envelope marker for *Plasmodium* spp.

## Conclusion

To build predictive models for malaria parasite multiplication we need to understand the dynamic relation between cycle time, cell volume and nuclear numbers. Therefore, we need to quantify those very basic cellular parameters in a time-resolved manner. The description of blood-stage development has been dominated by the useful, but limited, separation in ring, trophozoite and schizont stages. The more gradual change of key biophysical parameters, however, is best assessed by dynamic single cell analysis i.e. 4D live cell imaging, a technology that still has potential for a broader application in the malaria field (Gruring et al., 2011; De Niz et al., 2016). By verifying the dependency of merozoite number on schizogony duration we can establish whether timing plays a determinant role. This assumes that we also assess the variability in nuclear division rates. A strict dependency on the concentration of specific extrinsic factors would support a counting mechanism. In this context it will be important to verify whether progeny number generated by a parasite is an inheritable, possibly epigenetic, trait. Investigating this requires tracking progeny number from one to the next generation. Whether this number correlates with final cell size is another relevant parameter. Finally, all these parameters should be linked to parasite multiplication rates to predict how they might correlate with disease severity. Knowing that multiplication rates can increase throughout culture adaptation, we further need to assess the validity of those cellular parameters in samples that have more recently been isolated from patients (Stewart et al., 2020). Ultimately, we advocate for the systematic assessment of merozoite number in *Plasmodium* mutants that develop a growth phenotype, so that we can expand our list of candidate proteins implicated in defining progeny numbers and advance our understanding of malaria parasites proliferation mechanisms.

## Data Availability Statement

The original contributions presented in the study are included in the article/supplementary material. Further inquiries can be directed to the corresponding author.

## Author Contributions

Manuscript was written by JG with the help of VS and CS. Experimental data and **Figure 1** were generated by CS and VS. Data analysis was assisted by VS and JG. **Figure 2** was generated by VS with the help of JG. All authors contributed to the article and approved the submitted version.

## Funding

Funded by the Human Frontiers Science Program (CDA00013/2018-C), and the German Research Foundation (DFG) (349355339) to JG.

## Conflict of Interest

The authors declare that the research was conducted in the absence of any commercial or financial relationships that could be construed as a potential conflict of interest.
